# Convallatoxin enhance the ligand-induced mu-opioid receptor endocytosis and attenuate morphine antinociceptive tolerance in mice

**DOI:** 10.1038/s41598-019-39555-x

**Published:** 2019-02-20

**Authors:** Po-Kuan Chao, Hsiao-Fu Chang, Li-Chin Ou, Jian-Ying Chuang, Pin-Tse Lee, Wan-Ting Chang, Shu-Chun Chen, Shau-Hua Ueng, John Tsu-An Hsu, Pao-Luh Tao, Ping-Yee Law, Horace H. Loh, Shiu-Hwa Yeh

**Affiliations:** 1 Institute of Biotechnology and Pharmaceutical Research, National Health Research Institutes, Zhunan, 35053 Taiwan; 2 The PhD Program for Neural Regenerative Medicine, Taipei Medical University, Taipei, 110 Taiwan; 3 Cellular Pathobiology Section, Intramural Research Program, National Institute on Drug Abuse, NIH/DHHS, Baltimore, MD 21224 USA; 4 Center for Neuropsychiatric Research, National Heath Research Institutes, Zhunan, 35053 Taiwan; 5 Department of Pharmacology, Medical School University of Minnesota, Minneapolis, MN 55455-0217 USA

## Abstract

Morphine is a unique opioid analgesic that activates the mu-opioid receptor (MOR) without efficiently promoting its endocytosis that may underlie side effects. Our objective was to discover a novel enhancer of ligand-induced MOR endocytosis and determine its effects on analgesia, tolerance and dependence. We used high-throughput screening to identify convallatoxin as an enhancer of ligand-induced MOR endocytosis with high potency and efficacy. Treatment of cells with convallatoxin enhanced morphine-induced MOR endocytosis through an adaptor protein 2 (AP2)/clathrin-dependent mechanism, attenuated morphine-induced phosphorylation of MOR, and diminished desensitization of membrane hyperpolarization. Furthermore, co-treatment with chronic convallatoxin reduced morphine tolerance in animal models of acute thermal pain and chronic inflammatory pain. Acute convallatoxin administration reversed morphine tolerance and dependence in morphine-tolerant mice. These findings suggest convallatoxin are potentially therapeutic for morphine side effects and open a new avenue to study MOR trafficking.

## Introduction

Opioids have long been used to treat severe pain^1^. However, long-term use leads to tolerance, dependence and addiction^2^. No currently available drugs can completely substitute for opioids in most clinical opioid indications, and no treatment paradigms can successfully prevent the development of tolerance and addiction. Opioids primarily activate three G protein-coupled receptors (GPCRs) of the G_i_ subtype: the mu-, delta-, and kappa-opioid receptors (MOR, DOR, and KOR). Although the mechanisms of opioid-induced analgesia are not well-defined, it is now clear that activated opioid receptors are able to utilize both G-protein-dependent and G-protein-independent signaling pathways^3^. Furthermore, it is generally believed that opioid analgesics mainly exert their pharmacological effects by acting at the MOR^4^.

Compared to the full agonist D-ala2-nmephe4-gly-ol-enkephalin (DAMGO) and other high-efficacy opioids, such as etorphine and fentanyl^5^, morphine, the most commonly used opioid, has a poor ability to induce MOR endocytosis^6^. Previous studies indicated that a mutant recycling MOR (RMOR) that underwent endocytosis after morphine treatment was associated with reduced tolerance and cyclic AMP (cAMP) superactivation, a cellular hallmark of withdrawal, *in vitro*^7^. Compared to wild-type mice, RMOR knock-in mice showed a potentiated analgesic effect but less tolerance and withdrawal in response to morphine, indicating a beneficial effect of MOR internalization in morphine analgesia^8^. Furthermore, cocktails of morphine and a low dose of DAMGO^9^ or methadone^10^, which are MOR agonists with substantial MOR internalization ability, diminished both morphine tolerance and dependence in rats. However, it is hard to investigate the relationship between MOR endocytosis and morphine tolerance/dependence by two agonists mixture, due to their controversial mode of action in analgesia^11–15^. Therefore, it is worth developing an alternative strategy to specific upregulate MOR endocytosis in response to morphine to reduce tolerance and dependence. In this study, we established a high-throughput screening assay to identify non-opioid small-molecule enhancers of MOR endocytosis and validated the identified molecules in both cell cultures and animals.

## Materials and Methods

### Animals

Male wild-type C57BL/6 (B6) mice (25–30 g) and MOR-KO mice^16^ (provided by Dr. Pao-Luh Tao, National Health Research Institutes, Taiwan) were kept in a temperature-controlled animal room with a 12:12 h light-dark cycle. The protocol was approved by the Institutional Animal Care and Use Committee of the National Health Research Institutes, Taiwan. Animal experiments were conducted in accordance with the Policies on the Use of Animals in Neuroscience Research and the ethical guidelines for investigations of experimental pain in conscious animals established by the International Association for the Study of Pain.

### shRNA transfection

The pLKO.1 vectors encoding shRNA directed against the Na^+^/K^+^-ATPase α1 subunit (Clone ID: TRCN0000332624), clathrin heavy polypeptide (Clone ID: TRCN0000113160), AP2 α1 subunit (Clone ID: TRCN0000065112), and the universal negative control (Clone ID: TRCN0000231759) were from the National RNAi Core Facility (Institute of Molecular Biology/Genomic Research Center, Academia Sinica, Taiwan). Cells were transfected with the shRNA using TurboFect transfection reagent (Thermo Scientific). Twenty-four hours after transfection, cells were harvested for the internalization assay or membrane potential assay, and protein levels were determined using western blotting.

### Antibodies

The mouse monoclonal anti-Na^+^/K^+^-ATPase α1 subunit antibody (ab7671) was purchased from Abcam. The rabbit monoclonal anti-MOR antibody (NBP1-96656) was purchased from Novus Biologicals. The rabbit monoclonal anti-AP2 α antibody (GTX62588) and mouse monoclonal anti-clathrin heavy chain antibody (GTX22731) were purchased from GeneTex. The rabbit polyclonal anti-phospho-MOR Serine 375 antibody (#3451) was purchased from Cell Signaling Technology. The mouse monoclonal anti-β-actin antibody was purchased from Sigma. Alexa488-conjugated WGA and Alexa568-conjugated goat anti-rabbit IgG antibody were purchased from Invitrogen.

### Live cell imaging

CHO-K1 cells were grown in F12 medium containing 10% fetal bovine serum, 100 units/ml penicillin, and 100 μg/ml streptomycin in T-175 tissue culture flasks and harvested with trypsin/EDTA solution. Cells were transiently transfected with the human MOR-eGFP plasmid using NEPA21 electroporator gene transfection system (Nepa Gene) and subsequently seeded in a glass-bottom culture dish (MatTek Corporation). The poring pulse conditions for electroporation were as follows: 125 V, pulse length of 7.5 ms, inter-pulse intervals of 50 ms, and a 10% decay rate with plus polarity. The transfer pulse conditions were as follows: 20 V, 50-ms pulse length, 50-ms pulse interval, and a 40% decay rate with plus and minus polarities. After 24 h, cells were serum-starved for 3 h before MOR trafficking was measured. Cells were treated with vehicle, morphine (1 μM), convallatoxin (1 μM), morphine (1 μM) + convallatoxin (1 μM), or DAMGO (0.1 μM) for 30 min, and images were captured with a laser confocal microscope (TCS SP5II, Leica) using the same gain and exposure time for each group and then superimposed to determine the localization of MORs.

### Immunostaining

B6 mice were first injected subcutaneously (s.c.) with vehicle, morphine (10 mg/kg), convallatoxin (0.5 mg/kg), or morphine (10 mg/kg) + convallatoxin (0.5 mg/kg), or injected intrathecally (i.t.) with DAMGO (5 nmol), for 60 min, and then anesthetized by isoflurane and perfused transcardially with 1 × PBS followed by 4% paraformaldehyde. The DRG neurons were removed and postfixed for 12 h in 4% paraformaldehyde and then cryoprotected in 20% glycerol for 6 h at 4 °C. The DRG sections were glued to the platform of a Vibroslice tissue slicer. Transverse sections of 20-μm thickness were cut and the appropriate slices from each group were placed on the same microscope slide and processed identically during a standard immunofluorescence staining procedure. Double-labeling immunohistochemistry was performed as previously described^17^. Sections were first incubated with WGA conjugated with Alexa488 (1:1000) to label the plasma membrane. After washing, sections were incubated with permeabilization buffer (0.4% Triton X-100 and 2% FBS in PBS) for 1 h. MOR expression was detected by incubating sections in PBS with mouse anti-MOR antibody (1:300) for 24 h. Subsequently, sections were washed 4 times with the washing buffer (0.2% Triton X-100 in PBS) and then incubated in PBS with Alexa568-conjugated goat anti-rabbit antibody (1:200) and DAPI for 1 h. The slides were then washed 3 times with PBS and mounted with glycerol. Images were captured using a laser confocal microscope (TCS SP5II, Leica) and acquired using the same gain and exposure time.

### Internalization assay

The PathHunter GPCR internalization assay (DiscoveRx) was performed according to the manufacturer’s protocol. Briefly, PathHunter U2OS-MOR cells with complementary pieces of β-galactosidase genetically fused to the receptor and a component of the endocytic vesicle, respectively. In the present study, when activated MOR interacted with endosomes, the 2 fusion proteins formed a complete enzyme whose activity was detected by chemiluminescence. U2OS-MOR cells were grown to confluence in McCoy’s 5 A medium (GIBCO) containing 10% fetal bovine serum, 100 units/ml penicillin, 100 μg/ml streptomycin, 20 μg/ml G418 (Sigma), 5 μg/ml Hygromycin B (InvivoGen) and 25 mM HEPES in T-175 tissue culture flasks (Corning) and harvested with Cell Detachment Reagent (DiscoveRx). Cells (5,000 cells per well) were then seeded in black 384-well assay plates (Corning) with CP5 reagent (DiscoveRx) and incubated for 24 h before experiments. For high-throughput screening, cells were treated with 5 μl of HBSS, either containing morphine alone or in the presence of compounds (~5 mM in each well) from natural compound library plates (Greenpharma), at a final concentration of 0.3 μM and 5 μM, respectively. The effects of compounds on opioid-induced MOR endocytosis were determined by treating cells with HBSS, either containing various concentrations of ME or morphine alone or in the presence of compounds. The potency and efficacy of compounds in MOR endocytosis were determined in the absence or presence of the final concentration of ME (0.03 μM; ~EC_10_) or morphine (0.3 μM; ~EC_10_). Cells were incubated at room temperature for 1.5 h, followed by addition of 8 μl of PathHunter Detection kit (DiscoveRx) for 1 h, and analyzed for chemiluminescence on a Victor 2 plate reader (PerkinElmer). Experiments from each figure were run on the same day and using the same generation of U2OS-MOR cells to ensure accurate comparisons of data.

### Cyclic adenosine monophosphate accumulation (cAMP assay)

HEK-MOR cells (provided by Dr. Ping-Yee Law, University of Minnesota, USA) were cultured in DMEM (GIBCO) supplemented with 10% FBS, 100 units/mL penicillin, 100 μg/mL streptomycin, 400 μg/mL G418, and 2 mM L-glutamine in T-175 tissue culture flasks and harvested with trypsin/EDTA solution (GIBCO). Cells were plated at 72,000 per well under 100 μL/well of DMEM in 96-well, solid-bottom, white plates (GIBCO) and under 50 μL/well of HBSS containing forskolin or 3-isobutyl-1-methylxanthine at final concentrations of 1 μM and 500 μM, respectively. After 30 min of incubation at room temperature, the concentration of cAMP was determined using a LANCE Ultra cAMP Assay kit (Perkin Elmer). Two hours later, plate fluorescence was measured using a Victor 2 plate reader with excitation at 330 nm and emission at 615 nm and 665 nm. The effects of compounds on MOR-mediated inhibition of cAMP production were determined by treating cells with various concentrations of morphine alone or in the presence of the compounds. The results were presented as percent inhibition of forskolin-stimulated cAMP accumulation: [1 − (cAMP_compounds/forskolin_/cAMP_forskolin_)] × 100%.

### Spinal cord surgery

To reduce clathrin and AP2 expression in the spinal cord and DRG neurons^18^, shRNA against clathrin or AP2 or universal negative control was dissolved in artificial cerebrospinal fluid (aCSF) and injected intrathecally (i.t.) into the adult mice spinal cord using Micro-Renathane implantation tubing (Braintree Scientific) inserted in the T11–T13 intervertebral disc. After injection, the NEPA21 electroporator gene transfection system (Nepa Gene) was used to deliver shRNA into cells through needle electrodes inserted between the L1 and L6 vertebrae. The poring pulse conditions for electroporation were as follows: 150 V, pulse length of 5 ms, inter-pulse intervals of 50 ms, and a 10% decay rate with plus polarity. The transfer pulse conditions were as follows: 20 V, 50-ms pulse length, 50-ms pulse interval, and a 40% decay rate with plus and minus polarities^17^. All behavior tests were conducted at least three days after surgery. We confirmed that mice received shRNA universal negative control injection had no significant tissue injury and behavior dysfunction.

### Tail-flick test

Drug-induced antinociception was evaluated using the Tail-Flick Analgesia Meter (Columbia Instruments). The basal tail-flick latency was recorded before treatment and test latencies were recorded 45, 90, and 180 min after administration of drugs. A cutoff time of 10 s was set to avoid tissue damage. The analgesic effect was defined as the difference between the test latency and the basal latency (test latency − basal latency) at each time point. The AUC value was obtained by calculating the area under the time–response curve of the analgesic effect after treatment of the drugs. The percentage of the maximum possible effect (% of MPE) was calculated according to the following equation: % of MPE = [(test latency − baseline latency) ÷ (10 − baseline latency)] × 100. The ED_50_ was determined by the up and down method. Briefly, a series of test levels was chosen with equal spacing between each log of drug dose. Then, a series of trials (n ≧ 6) was performed following a rule that the dose was reduced after inhibition of the tail-flick response and the dose was increased if no inhibition of the tail-flick response was observed. Each mouse underwent only 1 trial. The ED_50_ value was derived from the equation ED_50_ = Xf + k × d, where Xf was the last dose administered, k was the tabular value, and d was the interval between doses.

For the chronic effects of compounds on the development of morphine tolerance, mice were chronically injected s.c. with vehicle, morphine (10 mg/kg), convallatoxin (0.5 mg/kg; 5% of the LD50)^19^, or morphine (10 mg/kg) + convallatoxin (0.5 mg/kg), twice daily for 8 days. The analgesic effect of each treatment was determined by testing the radiant heat tail-flick latency on day 1, and again after the final treatment on day 8. For acute effects of compounds in morphine tolerance, mice were first injected s.c. with morphine (10 mg/kg; twice daily for 8 days) to generate morphine-tolerant mice. At 12 h after the last morphine treatment, mice were administered s.c. with acute vehicle, morphine (10 mg/kg), convallatoxin (1 mg/kg), or morphine (10 mg/kg) + convallatoxin (0.5 or 1 mg/kg) to determine the radiant heat tail-flick latency at the indicated time points.

### Withdrawal jumping test

To determine the chronic effects of compounds on the development of morphine dependence, mice were chronically treated s.c. with vehicle, morphine (10 mg/kg), convallatoxin (0.5 mg/kg), or morphine (10 mg/kg) + convallatoxin (0.5 mg/kg) twice daily for 8 days. Morphine dependence was evaluated on day 9. To determine the acute effects of compounds in morphine-tolerant mice, mice received increasing doses of morphine (s.c.) for 4 days. On day 5, mice were acutely treated s.c. with convallatoxin (1 mg/kg), morphine (40 mg/kg), or morphine (40 mg/kg) + convallatoxin (0.5 or 1 mg/kg). Withdrawal jumping was precipitated by injecting each mouse with the opioid antagonist naloxone (1 mg/kg, s.c.) 2 h after the final administration of drugs. Each mouse was placed in an acrylic box (10 cm in diameter, 30 cm high), and the number of jumps was recorded for 30 min.

### CFA-induced chronic pain and Von Frey hair test

Chronic inflammatory pain was induced by CFA intraplantar injection. Briefly, each B6 mouse was injected with either saline or CFA (20 μl) in the footpad of the right hind paw under isoflurane anesthesia on post-inoculation day (PID) 0. From PID14 to 18, saline- and CFA-injected mice were twice daily administered s.c. with vehicle, morphine (10 mg/kg), convallatoxin (0.5 mg/kg), or morphine (10 mg/kg) and convallatoxin (0.5 mg/kg) combined. Mechanical allodynia was subsequently evaluated using von Frey filaments (range 0.1–1 g; IITC Life Science). Mice were placed on a mesh floor with 5 × 5 mm holes, covered with a cup to prevent visual stimulation, and allowed to adapt for 1 h prior to testing. On the test days, withdrawal responses following hind paw stimulation were measured, and mechanical allodynia was defined as changes in the amount of pressure required to induce withdrawal. The filaments were applied to the middle of the plantar surface of the hind paw.

### Statistical analysis

All mouse and *in vitro* experiments were repeated multiple times as indicated in the figure legends. Data are presented as the mean ± SEM from multiple individual experiments or as the mean ± sd performed at least in triplicate. Multiple groups were compared using 2-way ANOVA with Bonferroni’s *post hoc* tests or 1-way ANOVA with Newman–Keuls *post hoc* tests in Prism v. 5.0 software (GraphPad). The comparison of threshold between two groups, a Student’s *t*-test was used. *P* ≤ 0.05 was considered to be statistically significant.

## Results

### Convallatoxin augment opioid-induced MOR endocytosis

**Figure 1:**
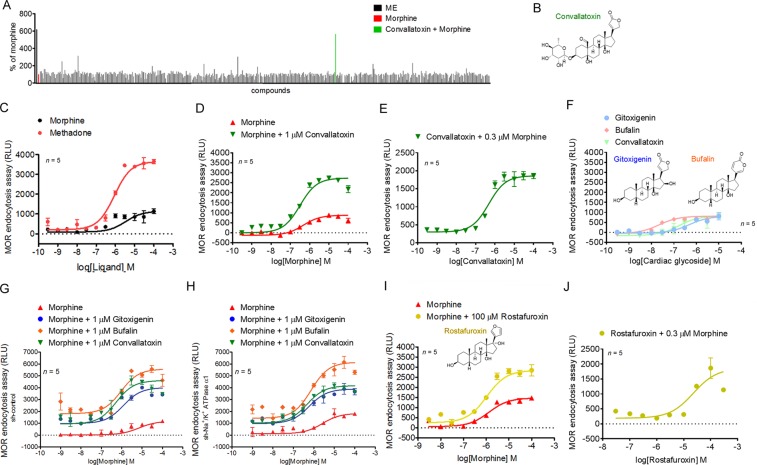
Convallatoxin modulate MOR endocytosis independently of Na^+^/K^+^-ATPase. (**A**) High-throughput screen to identify enhancers of MOR endocytosis. Results of the primary screen showing significant MOR endocytosis in convallatoxin + morphine treatment. Data are presented as percentages of the values for morphine (0.3 μM; ~EC_10_) alone. (**B**) Chemical structure of convallatoxin. (**C**) Concentration-response curves for morphine and methadone induction of MOR endocytosis. (**D**) Concentration-response curves for morphine induction of MOR endocytosis in the presence or absence of convallatoxin. (**E**) Concentration-response curves of convallatoxin in morphine mediated MOR endocytosis. (**F**) The effects of cardiac glycosides on MOR endocytosis are determined by treating U2OS-MOR cells with various concentrations of gitoxigenin, bufalin, and convallatoxin. Chemical structure of bufalin, and gitoxigenin. (**G**,**H**) Involvement of Na^+^/K^+^-ATPase in the effect of cardiac glycosides. U2OS-MOR cells were transiently transfected with sh-control (**G**) or sh-Na^+^/K^+^-ATPase α1 (**H**) for 24 h prior to MOR internalization assay. (**I**) Concentration-response curves for morphine-induced MOR endocytosis with or without rostafuroxin. (**J**) Concentration-response curves of rostafuroxin in morphine-mediated MOR endocytosis. RLU, relative light units. Data in **C**-**J**, MOR internalization was measured by an enzyme complementation assay in U2OS-MOR cells. All values indicate the mean ± SD.

To identify enhancers of ligand-induced MOR endocytosis, we screened a natural compound library (480 compounds) in the presence of morphine, using a sensitive enzyme complementation assay for MOR endocytosis in human osteosarcoma U2OS cells expressing human MOR (U2OS-MOR). [Met5]-enkephalin (ME) induced MOR internalization, serving as a positive control for the experimental system. Results were normalized to those in morphine-treated samples. Convallatoxin, that is capable of inhibiting of Na^+^/K^+^-ATPase^20^ were retrieved from the primary screens (Fig. 1A,B), and the potency and efficacy were tested. Compare to methadone, morphine is a MOR agonist with relatively weak ability to induce MOR endocytosis (Fig. 1C). Concentration-response curves for morphine-induced MOR endocytosis were obtained in the absence or presence of convallatoxin at 1 μM (Fig. 1D). However, convallatoxin significantly enhanced the E_max_ of morphine-induced MOR endocytosis (Fig. 1D). We then determined the potency and efficacy of convallatoxin in regulating ligand-induced MOR endocytosis in the presence of the effective concentration at 10% activation (EC_10_) of morphine (0.3 μM) obtained from Fig. 1D. Convallatoxin enhanced morphine-induced MOR endocytosis in a concentration-dependent manner (Fig. 1E). Additional cardiac glycosides, including gitoxigenin but not bufalin, though itself induced MOR endocytosis slightly at high concentration (Fig. 1F). To determine whether the enhancing effects of cardiac glycosides on morphine-induced MOR endocytosis was mediated through their inhibition of Na^+^/K^+^-ATPase, endogenous Na^+^/K^+^-ATPase expression was suppressed by short hairpin RNA (shRNA) against the Na^+^/K^+^-ATPase α1 subunit (sh-Na^+^/K^+^-ATPase α1) in U2OS-MOR cells (Supplementary Fig. 1). No significant difference in the enhancing effects of convallatoxin was observed between cells transfected with the universal negative control shRNA (sh-control) and sh-Na^+^/K^+^-ATPase α1 (Fig. 1G,H). Furthermore, treatment of U2OS-MOR cells with rostafuroxin, a cardiac glycoside without Na^+^/K^+^-ATPase inhibitory activity^21^, also enhanced morphine efficacy (β-factor) by 2-fold (Fig. 1I,J). These results indicate that convallatoxin potentiate morphine-induced MOR endocytosis through a Na^+^/K^+^-ATPase-independent mechanism.

### Convallatoxin regulate MOR endocytosis through an AP2/clathrin-dependent mechanism

**Figure 2:**
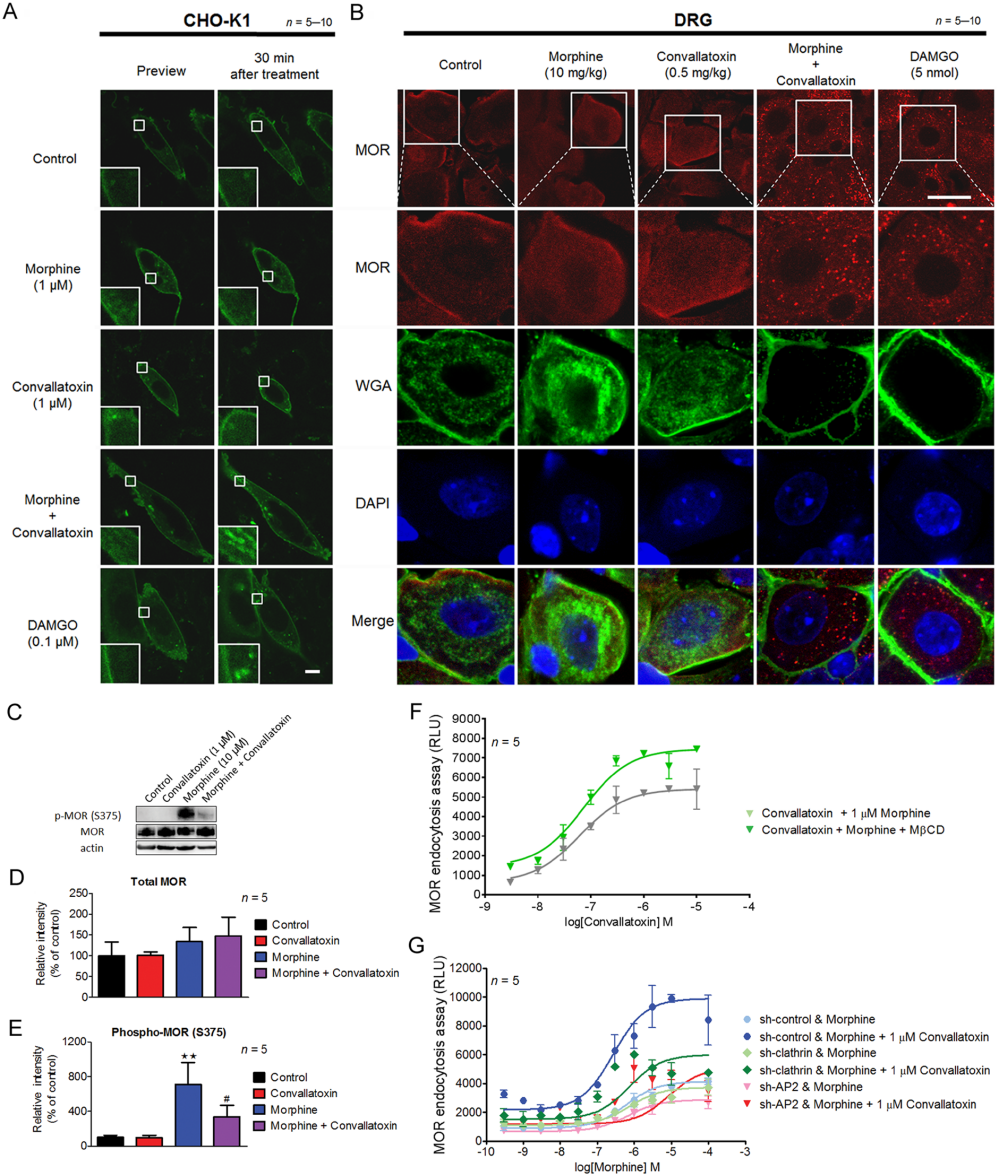
Effect of convallatoxin on opioid–induced MOR endocytosis. (**A**) Representative live cell imaging of the distribution of MOR-eGFP in CHO-K1 cells before and 30 min after drug treatment using a real-time confocal microscopy. Scale bars, 10 μm. (**B**) Representative immunofluorescence images of the distribution of MOR (red) and WGA (green) in the mouse DRG 1 h after drug treatment. The localization of MOR and WGA-labeled plasma membrane was monitored by confocal microscopy. DAPI (blue) was used as a nuclear marker. Scale bar, 20 μm. (**C**) Convallatoxin attenuated morphine-induced MOR phosphorylation. HEK-MOR cells were treated as indicated for 30 min. Phosphorylation of MOR at serine 375 (**C**,**D**) and total MOR expression (**C**,**E**) were analyzed by western blotting. Protein expression was quantified using densitometry (**D**,**E**). (**D**) *F*_3,12_ = 7.36, *p* < 0.01; (**E**) *F*_3,12_ = 0.08, *p* > 0.05 (1-way ANOVA). **p < 0.01 versus vehicle control group. ^#^*p* < 0.05 versus morphine group (Newman-Keuls *post hoc* tests). (**F**) Concentration-response curves of convallatoxin in morphine-induced MOR endocytosis in the presence or absence of MβCD. Data are percentages of the values for morphine (0.3 μM; ~EC_10_) alone. (**G**) Silencing of AP2 and clathrin attenuated the effect of convallatoxin on morphine-induced MOR endocytosis. U2OS-MOR cells were transiently transfected with sh-control, sh-clathrin or sh-AP2 for 24 h, prior to MOR internalization assay. All values indicate the mean ± SD. RLU, relative light units.

We further examined the enhancement of morphine-induced MOR endocytosis of convallatoxin, a BBB-penetrating cardiac glycoside^22^, using live-cell imaging in Chinese hamster ovary (CHO)-K1 cells expressing MOR-eGFP. DAMGO induced MOR internalization, serving as a positive control for the experimental system. Convallatoxin significantly augmented morphine-induced MOR internalization (Fig. 2A), whereas morphine or convallatoxin alone had no effect. Additionally, we observed similar results *in vivo* by immunofluorescent staining for MOR and the plasma membrane marker, wheat germ agglutinin (WGA), in dorsal root ganglion (DRG) neurons obtained from mice co-treated with morphine and convallatoxin (Fig. 2B). Thus, here we first validated that convallatoxin is a unique enhancer of opioid-induced MOR endocytosis.

In addition, we evaluated the ability of convallatoxin to alter other MOR-mediated responses, including G protein-dependent signaling (inhibition of adenylyl cyclase and activation of G protein-coupled inwardly rectifying potassium (GIRK) channels) and G protein-independent signaling (MOR phosphorylation by GPCR kinase (GRK)). Convallatoxin only slightly attenuated morphine-induced inhibition of cAMP production using cAMP assay in human embryonic kidney 293 (HEK-293) cells constitutively expressing human MOR (HEK-MOR; Supplementary Fig. 2).

Serine 375 of the MOR is a primary phosphorylation site for GRK responsible for MOR desensitization that is involved in the development of opioid tolerance^23^. After activation by morphine, MOR exhibits selective and persistent phosphorylation at this site both *in vitro* and *in vivo*^24,25^. We observed that pretreatment of convallatoxin attenuated morphine-induced phosphorylation at serine 375 in HEK-MOR cells (Fig. 2C,E). Expression of MOR protein was not affected by either convallatoxin or morphine. Thus, convallatoxin decreased morphine-mediated MOR phosphorylation without altering protein expression. On the other hand, pretreatment of the cholesterol-depleting endocytosis inhibitor methyl-β-cyclodextrin (MβCD)^26^ or silencing two genes well-involved in receptor endocytosis, adaptor protein 2 (AP2) and clathrin, attenuated the effects of convallatoxin (Fig. 2F,G). Thus, convallatoxin may enhanced morphine-induced MOR endocytosis in an AP2/clathrin-dependent manner.

### Convallatoxin regulates morphine-mediated GIRK channel activation in mouse pituitary AtT-20 cells

**Figure 3:**
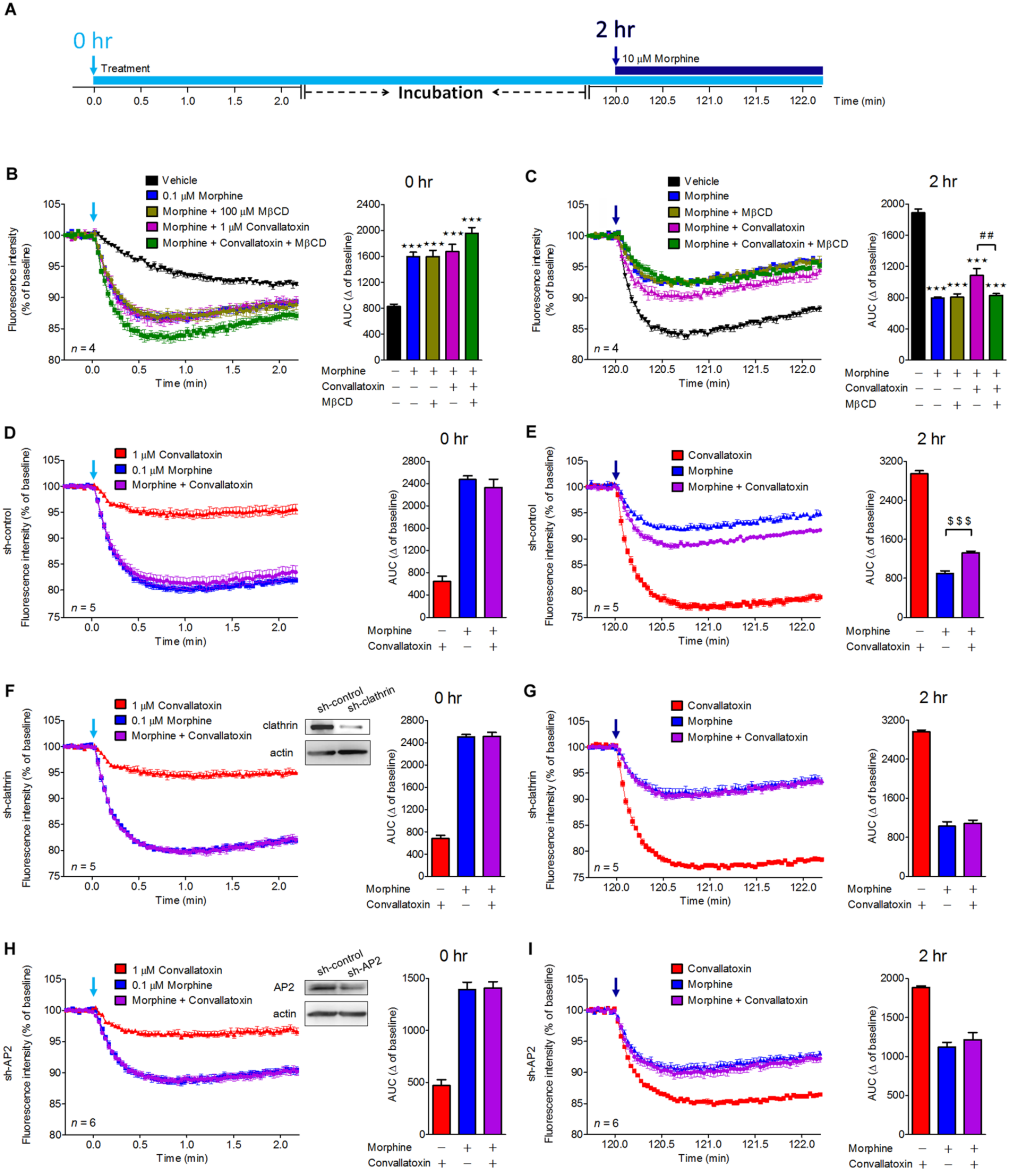
Effects of convallatoxin on morphine-mediated membrane potential hyperpolarization in MOR-expressing AtT-20 cells. (**A**) Flowchart showing experiments testing the effects of convallatoxin on morphine activation of GIRK channels. AtT-20 Cells were transfected with myc-MOR expression plasmid for 24 h prior to all membrane potential assays. (**B**) Acute effects of each treatment in MOR-expressing AtT-20 cells were measured using membrane potential assay. AUC: *F*_4,15_ = 23.97, *p* < 0.0001 (1-way ANOVA). (**C**) After 2 h of incubation, chronic effects of each treatment on MOR desensitization were determined by rechallenging cells with 10 μM morphine. AUC: *F*_4,15_ = 84.32, *p* < 0.0001 (1-way ANOVA). (**D**,**F**,**H**) AtT-20 Cells were co-transfected with myc-MOR and sh-control (**D**), sh-clathrin (**F**), or sh-AP2 (**H**) for 24 h prior to membrane potential assay. Silencing of clathrin (**F**) and AP2 (**H**) did not attenuate the acute effect of convallatoxin. Immunoblot showing expression of clathrin (F) or AP2 (H) in clathrin- or AP2-knockdown AtT-20 cells (upper panel). AUC: (**D**) *F*_2,12_ = 88.35; (**F**) *F*_2,12_ = 323.2; (**H**) *F*_2,15_ = 75.45; all *p* < 0.001 (1-way ANOVA). (**E**, **G**, **I**) Both clathrin (**I**) and AP2 (**G**) were involved in the chronic convallatoxin effect. AUC: (**E**) *F*_2,12_ = 458.8; (**G**) *F*_2,12_ = 287.2; (**I**) *F*_2,15_ = 42.45; all *p* < 0.001 (1-way ANOVA). ***p < 0.001 versus vehicle group. ^##^*p* < 0.01 versus morphine + convallatoxin group. ^$$$^*p* < 0.001 versus morphine-alone group (Newman-Keuls *post hoc* tests). All values indicate the mean ± SD.

Next, we determined whether convallatoxin have any effect on GIRK activation^27^, a G protein-dependent signaling pathway known to contribute to MOR-mediated analgesia^28,29^. AtT-20 cells endogenously expressing GIRK1/GIRK2 channels were transiently transfected with the myc-MOR expression plasmid prior to the entire membrane potential assays (Fig. 3A) and the area under the curve (AUC) was calculated to represent the total drug exposure integrated over time^17^. Acute treatment with morphine caused MOR-dependent membrane potential hyperpolarization. Convallatoxin co-treatment had no effect on the acute morphine-elicited response (Fig. 3B).

The continued presence of agonists can reduce the response to rechallenge with a subsequent high concentration of morphine, and this phenomenon is associated with clinical morphine tolerance. Chronic morphine treatment produced rapid desensitization of GIRK currents^30^. Therefore, we examined the role of convallatoxin in a cellular model of morphine tolerance by acutely rechallenging cells with morphine 2 h after chronic morphine treatment (Fig. 3A). Chronic morphine reduced the effect of a subsequent high concentration of morphine, however, the desensitization was attenuated by co-treatment with convallatoxin, with cells showing greater membrane potential hyperpolarization after morphine rechallenge (Fig. 3C). Furthermore, pretreatment with MβCD, or silencing AP2 and clathrin, significantly attenuated the effects of convallatoxin in response to chronic but not acute morphine (Fig. 3B,C, MβCD; Fig. 3D,E, sh-control; Fig. 3F,G, sh-clathrin; Fig. 3H,I, sh-AP2). This finding suggests that the regulation of receptor endocytosis by convallatoxin is necessary for attenuation of chronic morphine-mediated MOR desensitization.

### Convallatoxin treatment diminish morphine tolerance in a clathrin/AP2-dependent manner in mice

**Figure 4:**
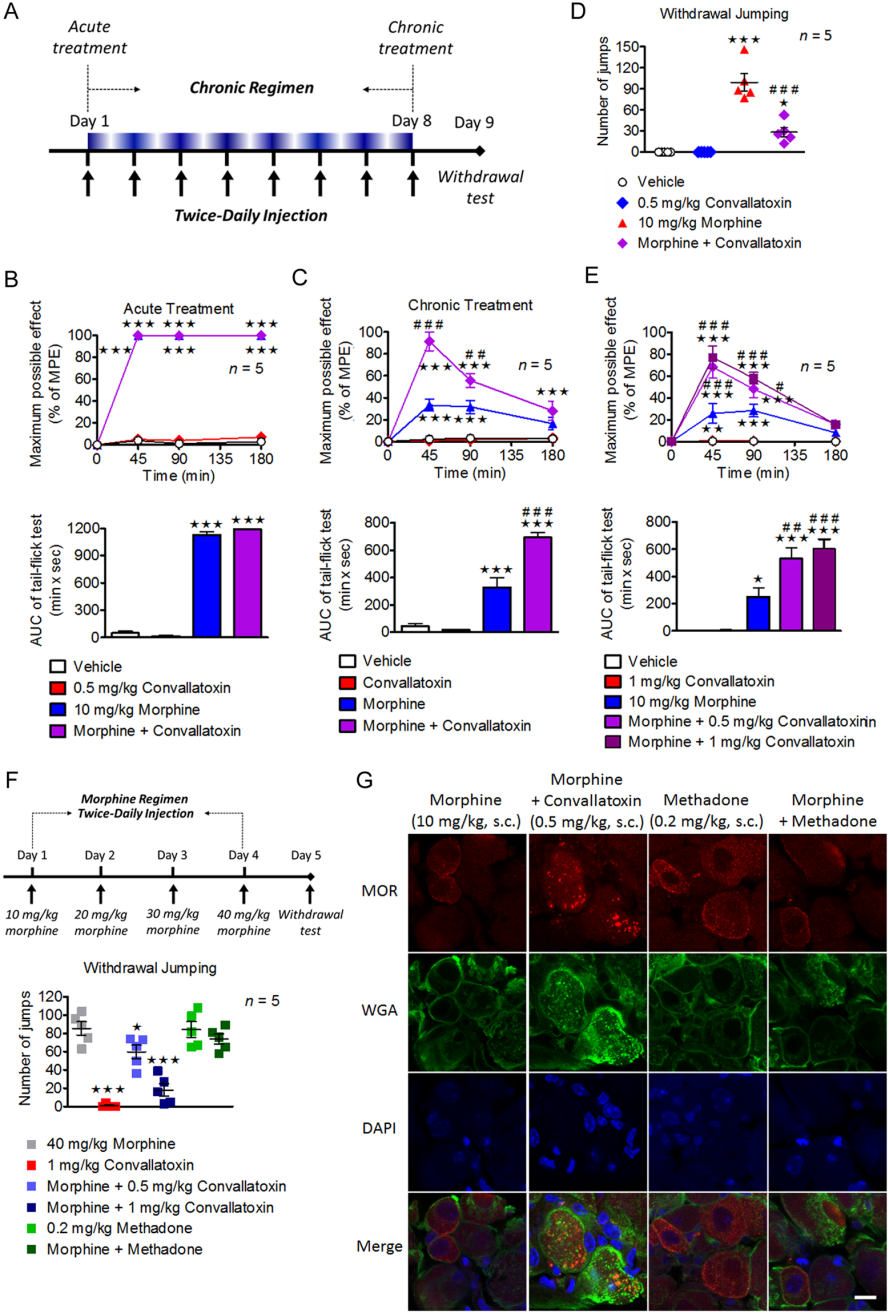
Involvement of convallatoxin in morphine antinociceptive tolerance and dependence. (**A**) Experiment flowchart for testing of drug effects on morphine tolerance and dependence. (**B**,**C**) Acute (**B**) and chronic (**C**) antinociceptive effects of each treatment in mice measured using tail-flick test. (**B**; upper panel) Treatment *F*_3,16_ = 1938, min *F*_3,48_ = 3828, interaction *F*_9,48_ = 1089; (**C**; upper panel) Treatment *F*_3,16_ = 80.31, min *F*_3,48_ = 41.5, interaction *F*_9,48_ = 18.41; all *p* < 0.001 (2-way ANOVA). Quantitative results from time-response curves are presented as AUC. (**B**; lower panel) *F*_3,16_ = 1068; (**C**; lower panel) *F*_3,16_ = 62.45; all *p* < 0.001 (1-way ANOVA). (**D**) Chronic convallatoxin diminished naloxone-precipitated withdrawal jumping. *F*_3,16_ = 44.58, *p* < 0.001 (1-way ANOVA). (**E**) The acute antinociceptive effects of each treatment in morphine-tolerant mice. Treatment *F*_4,20_ = 30.24, min *F*_3,60_ = 81.9, interaction *F*_12,60_ = 18.07; all *p* < 0.001 (2-way ANOVA; upper panel). Quantitative results from time-response curves, presented as AUC. *F*_4,20_ = 26.54, *p* < 0.001 (1-way ANOVA; lower panel). (**F**) Experiment flowchart determining the acute drug effects on naloxone-precipitated withdrawal jumping in morphine-tolerant mice (upper panel). Acute convallatoxin diminished morphine withdrawal. *F*_3,16_ = 38.56, *p* < 0.001 (1-way ANOVA; lower panel). (**G**) Representative immunofluorescence images of MOR (red) and WGA (green) distribution in the mouse DRG after chronic drug treatment. DAPI (blue) was a nuclear marker. Scale bar, 20 μm. Data in **B** (upper panel), **C** (upper panel), **E** (upper panel), **p < 0.01, ***p < 0.001 versus vehicle group. ^#^*p* < 0.05, ^##^*p* < 0.01, ^###^*p* < 0.001 versus morphine-alone group (Bonferroni’s *post hoc* tests). Data in **B** (lower paner), **C** (lower panel), (**D**,**E**) (lower paner), (**F**), *p < 0.05, ***p < 0.001 versus vehicle group. ^#^*p* < 0.05, ^##^*p* < 0.01, ^###^*p* < 0.001 versus morphine-alone group (Newman-Keuls *post hoc* tests). The values indicate the mean ± s.e.m. MPE, maximum possible effect.

To assess the effect of convallatoxin on morphine-produced antinociception, tail-flick tests after acute and chronic treatments were performed as shown in Fig. 4A. Acute morphine and morphine plus convallatoxin displayed a similar potency (ED_50_: morphine, 2.6 ± 0.3 mg/kg; morphine + convallatoxin, 2.8 ± 0.7 mg/kg) and magnitude of antinociception (Fig. 4B). However, chronic morphine plus convallatoxin resulted in greater antinociception (ED_50_: morphine, 10.6 ± 1.2 mg/kg; morphine + convallatoxin, 6.4 ± 1.2 mg/kg) (Fig. 4C), and more MOR endocytosis (Fig. 4G) relative to chronic morphine alone. The development of physical dependence was tested after chronic morphine treatment (Fig. 4A). Chronic co-administration of morphine plus convallatoxin showed reduced withdrawal jumping relative to chronic morphine alone (Fig. 4D). Thus, these results indicate that chronic treatment with convallatoxin reduces morphine tolerance in acute thermal pain and inhibits the development of physical dependence on morphine.

Tolerance and dependence may be unavoidable after long-term use of morphine^31^, and a strategy to reverse these characteristics should be of benefit for chronic pain management^32^. We first investigated the effects of acute convallatoxin administration in morphine-tolerant mice received chronic morphine injection twice daily for 8 days. On the test day, mice were challenged with morphine, either alone or in combination with convallatoxin, and tested for antinociception in tail-flick model. Morphine-tolerant mice received morphine with convallatoxin showed a significantly greater antinociceptive response than mice received morphine alone (Fig. 4E). Moreover, acute co-administration of convallatoxin attenuated naloxone-precipitated morphine withdrawal in morphine-tolerant mice (Fig. 4F). Although locomotor activity cannot be directly measured in the withdrawal jumping test, we did not observe a significant difference between study groups in the entire test. These results indicated that acute convallatoxin treatment was able to reverse morphine tolerance and withdrawal.

**Figure 5:**
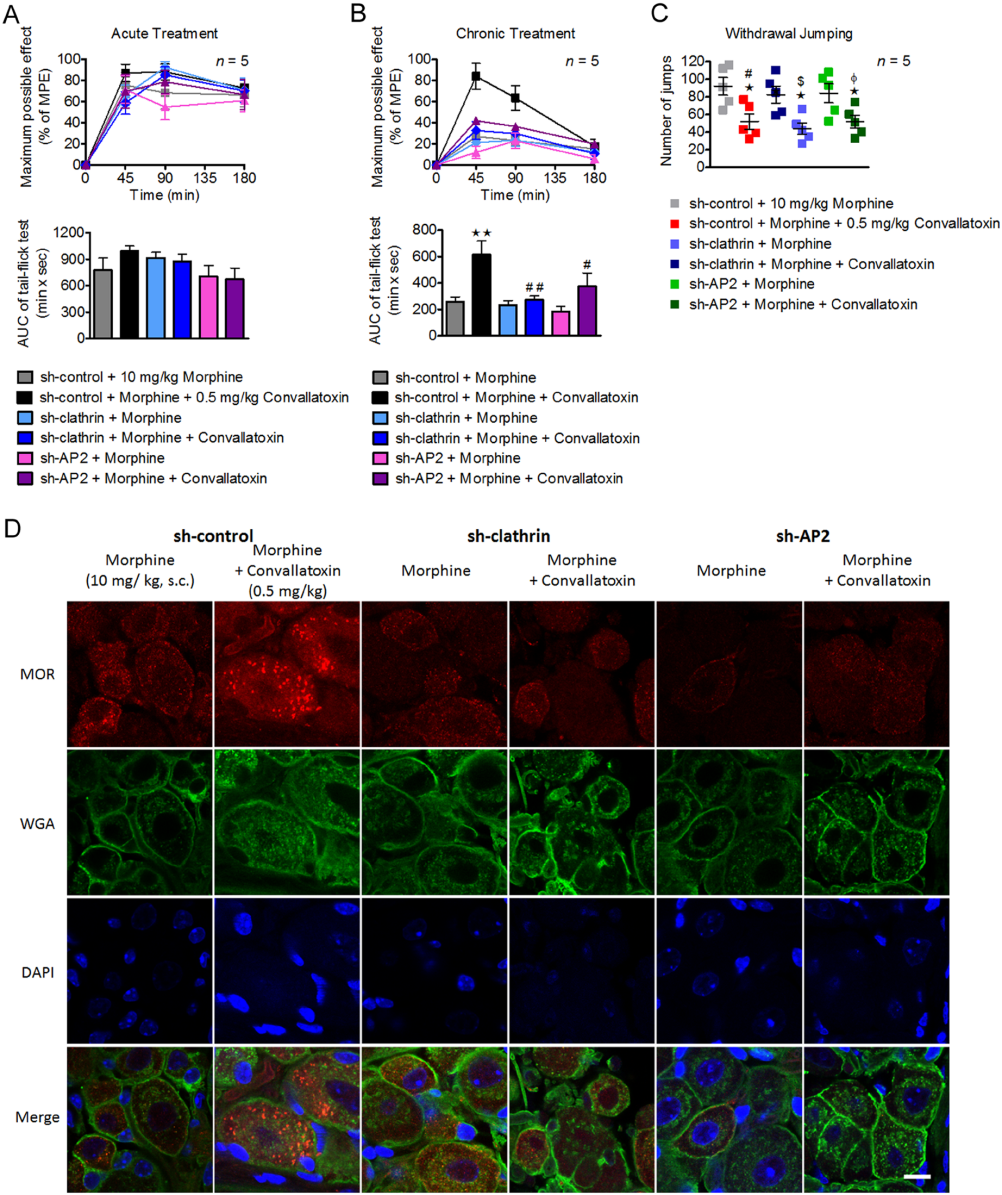
Down-regulation of clathrin and AP2 attenuated the effect of convallatoxin in morphine antinociceptive tolerance. (**A**,**B**) The sh-control, sh-clathrin, or sh-AP2 was delivered into the spinal cord of wild-type (WT) mice by using direct *in vivo* electroporation. Three days after surgery, the acute (**A**) and chronic (**B**) antinociceptive effects of each treatment were measured using a tail-flick test. (**A**; upper panel) Treatment *F*_5,24_ = 0.47, min *F*_3,72_ = 168.2, interaction *F*_15,72_ = 1.44; (**B**; upper panel) Treatment *F*_5,24_ = 6.9, min *F*_3,72_ = 72.56, interaction *F*_15,72_ = 5.81; all *p* < 0.001 (2-way ANOVA). Quantitative results from the upper panel of (**A**) and (**B**) are presented as AUC. (**A**; lower panel) *F*_5,24_ = 1.43, *p* > 0.05; (**B**; lower panel) *F*_5,24_ = 5.64, *p* < 0.01 (1-way ANOVA). (**C**) Chronic convallatoxin administration still diminished naloxone-precipitated withdrawal jumping in clathrin and AP2 knockdown mice. *F*_5,24_ = 5.29, *p* < 0.01 (1-way ANOVA). (**D**) Representative immunofluorescence images of MOR (red), WGA (green) for each treatment in mouse DRG neurons were visualized using immunostaining. DAPI (blue) was used as a nuclear marker. Scale bars, 20 μm. Data in **B** (upper paner), ***p < 0.001 versus sh-control + morphine group (Bonferroni’s *post hoc* tests). Data in **B** (lower paner), **C**,**D**, *p < 0.05, **p < 0.01, ***p < 0.001 versus sh-control + morphine group. ^#^*p* < 0.05, ^##^*p* < 0.01 versus sh-control + morphine + convallatoxin group. ^§^*p* < 0.05 versus sh-clathrin + morphine group. ^$^*p* < 0.05 versus sh-AP2 + morphine group (Newman-Keuls *post hoc* tests). Data are presented as the mean ± s.e.m. MPE, maximum possible effect.

Because convallatoxin enhance MOR endocytosis through clathrin/AP2 pathway, we examined whether clathrin/AP2 play any role of convallatoxin in morphine analgesia. Intrathecal electroporation of shRNA slienced either clathrin or AP2 in DRG neurons, without influence the basal nociceptive sensitivity of mice. However, the enhancement effect of chronic convallatoxin on morphine analgesia and MOR endocytosis were abolished in the spinal AP2 or clathrin knockdown mice (Fig. 5A,B,D), suggesting that clathrin AP2 adaptor complex are involved in the chronic effect of convallatoxin on morphine-mediated analgesia *in vivo*. In contrast, the morphine withdrawal symptoms were still influenced by convallatoxin in these knockdown mice (Fig. 5C), consistent with the previous study that supraspinal region, especially locus coeruleus (LC) is important in morphine withdrawal^33^, however, the endocytotic proteins in this area were not influenced by intrathecal electroporation.

### Convallatoxin treatment reduces morphine tolerance in mice with chronic inflammatory pain

**Figure 6:**
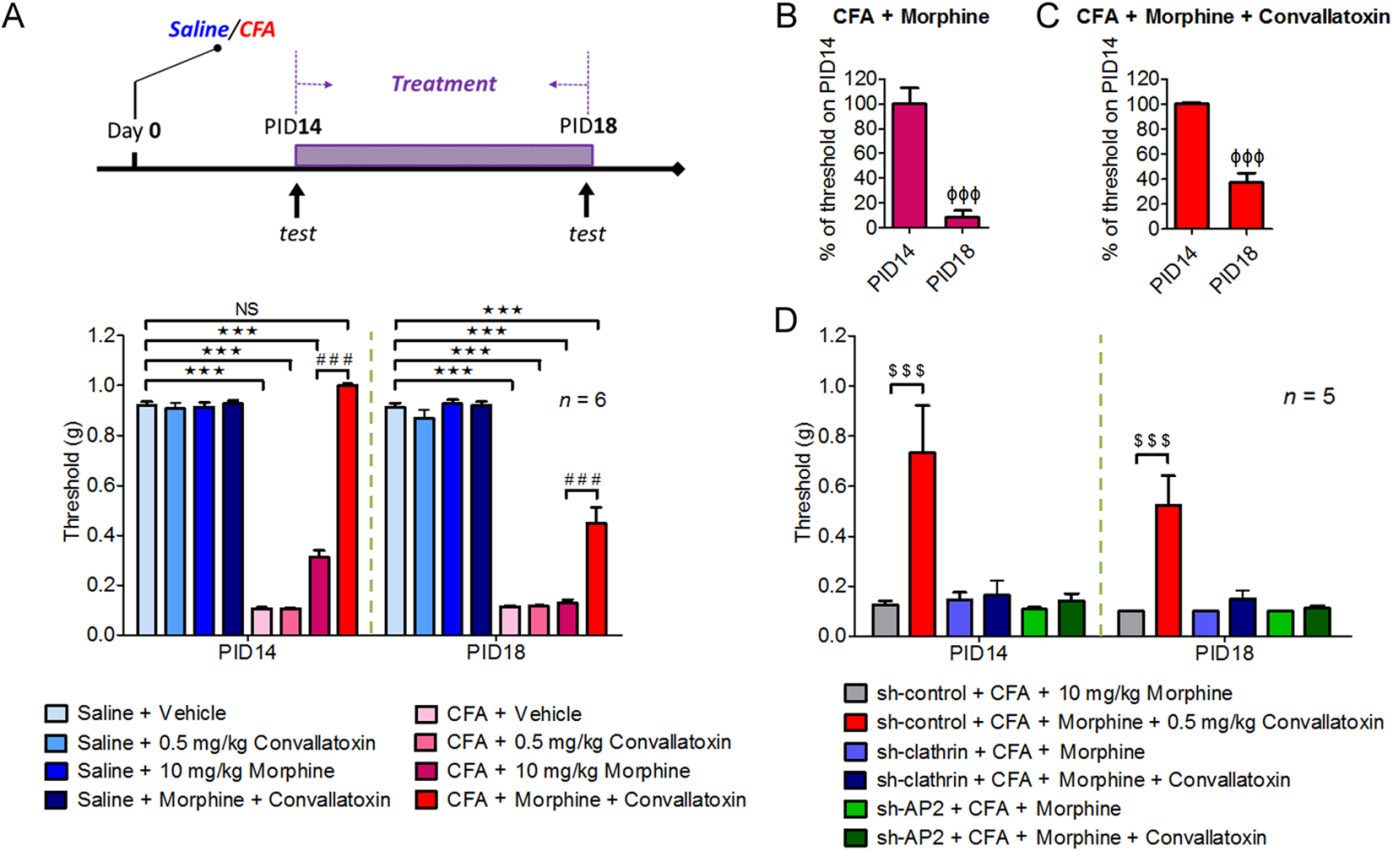
Convallatoxin alters morphine-induced alleviation of mechanical allodynia through clathrin and AP2 in the CFA-induced arthritic mouse model. (**A**) Experimental flowchart for effects of convallatoxin on allodynia. Mice receive an intraplantar injection of saline or CFA to induce local inflammation. Allodynia, expressed in g, is evaluated in CFA- and saline-treated mice 45 min after the last drug injection on post-inoculation days (PIDs) 14, and 18. Treatment *F*_7,40_ = 589.4, time *F*_1,40_ = 65.54, interaction *F*_7,40_ = 34.96; all *p* < 0.001 (2-way ANOVA). (**B**,**C**) Quantitative results from (**A**) are presented as percentages of the threshold on PID 14 for CFA + morphine (**B**) or CFA + morphine + convallatoxin (**C**). (**B**,**C**) all *p* < 0.001 (student’s *t*). (**D**) Silencing *clathrin* or *AP2* attenuates the effect of convallatoxin on morphine antinociception. The mouse spinal cord was electroporated with *sh-control*, *sh-clathrin*, or *sh-AP2* on PID 7. Treatment *F*_5,24_ = 58.27, time *F*_1,48_ = 1.32, interaction *F*_5,48_ = 0.58; all *p* < 0.001 (2-way ANOVA). Data in (A, lower panel) and (D): ***p < 0.001 versus saline + vehicle group; ^###^*p* < 0.001 versus CFA + morphine group; ^$$$^*p* < 0.001 versus *sh-control* +CFA + morphine group (Bonferroni’s post hoc test). Data in B and C, right panel: ^ΦΦΦ^*p* < 0.001 versus threshold of each group on PID 14; ^$$$^*p* < 0.001 versus *sh-control* + CFA + morphine group (Newman-Keul’s post hoc test).

Opioids are used to manage chronic osteoarticular pain, but repeated administration develops analgesic tolerance^34^. We then investigated whether convallatoxin exerts beneficial effects on morphine analgesia in the complete Freund’s adjuvant (CFA)-induced rheumatoid arthritis model in mouse. It was previously shown that allodynia is almost maximal 2 weeks after intraplantar injection of CFA^35^, and we used this treatment paradigm accordingly. Mice were injected with each treatment twice-daily on post-inoculation days (PIDs) 14 to 18, and mechanical allodynia was measured by the Von Frey test to examine analgesic effects of each treatment on PIDs 14, 16 and 18 (Fig. 6A). The threshold of mechanical allodynia in CFA-treated mice decreased significantly during PIDs 14 to 18 compared to saline-treated mice and reached a plateau (Fig. 6A)^35^. In CFA-treated mice, acute treatment with morphine + convallatoxin, but not with morphine alone, on PID 14 diminished mechanical allodynia, and the paw withdrawal threshold was similar to that in non-CFA-treated groups (Fig. 6A). Furthermore, the morphine plus convallatoxin group showed reduced analgesic tolerance relative to the morphine group. After daily treatment of morphine or morphine + convallatoxin for continuous 5 days (PID18), the threshold of mechanical allodynia of these two experimental groups was reduced by approximately 92% (Fig. 6B) and 67% (Fig. 6C), respectively, in CFA-treated mice, whereas convallatoxin itself did not produce any analgesic effect. Morever, silencing of clathrin and AP2 in DRG neurons of B6 mice significantly decreased the effect of convallatoxin on both acute and chronic morphine analgesia (Fig. 6D), further supporting that convallatoxin enhanced morphine efficacy and attenuated the development of analgesic tolerance through clathrin/AP2-dependent manner in chronic inflammatory pain.

## Discussion

**Figure 7:**
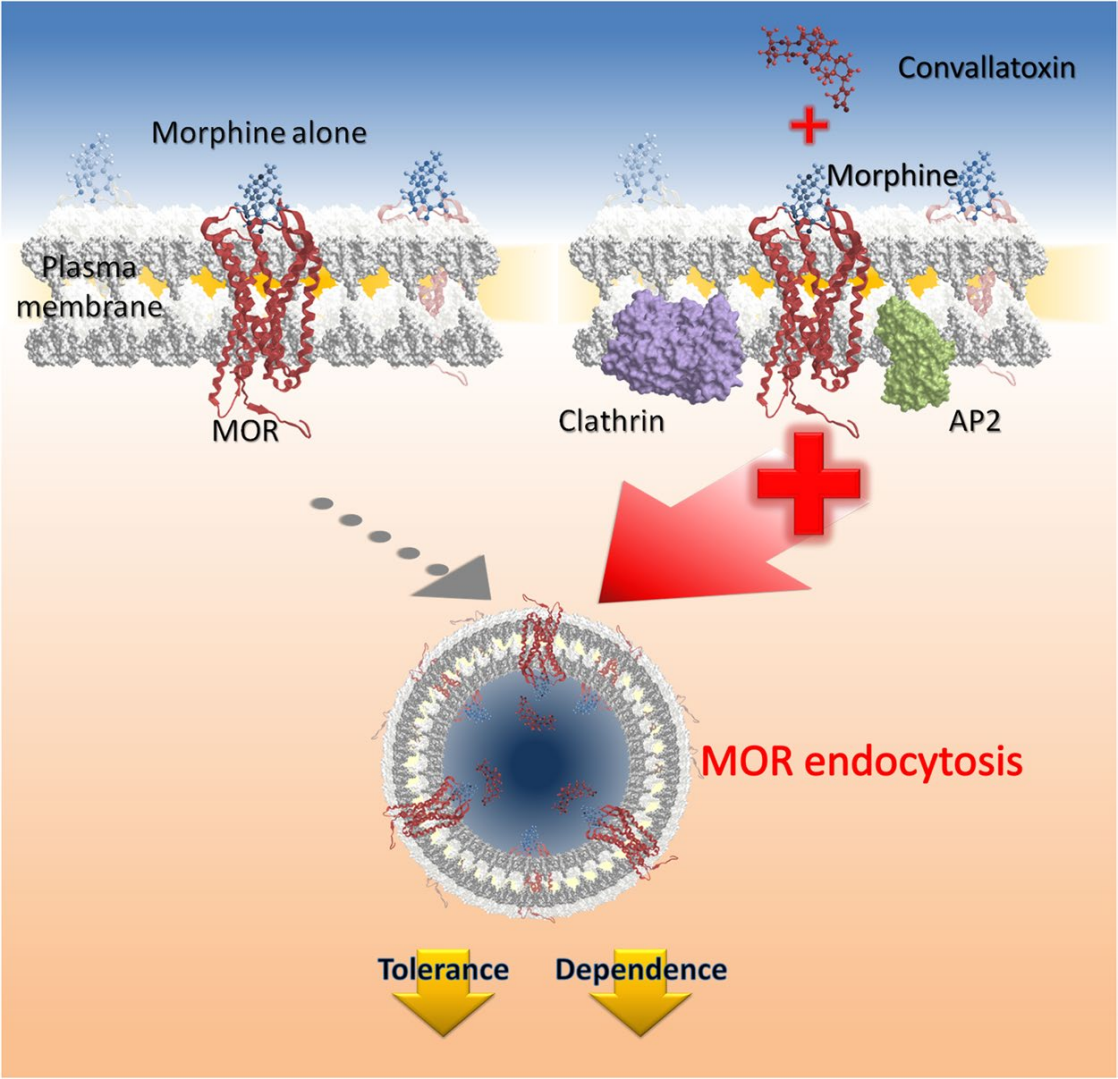
Convallatoxin is enhancer of morphine-mediated mu opioid receptor endocytosis. Convallatoxin could be a small-molecule, positive allosteric modulators for morphine-induced MOR endocytosis. Morphine binds to MOR and elicits antinociceptive effects. However, its poor ability to induce MOR endocytosis results in morphine tolerance after long-term use. Convallatoxin promotes better induction of receptor endocytosis by morphine, accompanied by better antinociception and reduced morphine tolerance.

Cardiac glycosides, such as convallatoxin, are used to treat cardiac failure and atrial fibrillation through inhibition of Na^+^/K^+^-ATPase^36^. Besides acting as Na^+^/K^+^-ATPase inhibitor, convallatoxin may regulate other proteins to potentially serve as therapeutic agents. Cardiac glycoside selectively reduces the protein levels and intrinsic transcriptional activity of steroid receptor coactivator (SRC)-1 and SRC-3 and it can be a potentially broad-spectrum inhibitor for cancer^37^. Cardiac glycoside is also shown to inhibit interferon-β expression and tumor necrosis factor signaling, that can be a proposed treatment for inflammatory and autoimmune diseases respectively^38^. In this study, we first identified convallatoxin as a novel enhancer of morphine-induced MOR endocytosis (Fig. 1) independently of Na^+^/K^+^-ATPase inhibition (Fig. 1G–J). Furthermore, convallatoxin prevented the development or expression of morphine tolerance and withdrawal in a MOR-dependent manner in animals (Figs 4–5), and enhanced morphine-produced analgesia in mice with CFA-induced hind paw allodynia (Fig. 6). Our results demonstrate that convallatoxin can modulate both opioid-induced MOR endocytosis and analgesia through MOR-mediated signaling pathway (Fig. 7).

Cardiac glycosides induced slight MOR endocytosis in U2OS-MOR cells (Fig. 1F), but not in CHO-K1 cells or DRG neurons (Fig. 2A,B). This inconsistency may have been related to the different sensitivity of each assay, or to cell-type-specific effects of these drugs on U2OS-MOR cells^39^. By contrast, significant MOR endocytosis was observed when cells were co-treated with cardiac glycosides and morphine in both the MOR internalization assay and the immunofluorescence assay. Upon agonist-induced activation of MOR, β-arrestin-2 recruitment is believed to promote receptor internalization through linkage of GPCRs to proteins of the endocytotic machinery, including clathrin^40^ and AP2^41^. However, in some cases, recent research has shown that β-arrestin-2 recruitment is not necessary for internalization of GPCRs. Several neuronal GPCRs, including the metabotropic glutamate receptor 1^42^, serotonin 5-HT2A receptor^43^ and M2 muscarinic cholinergic receptor^44^, show β-arrestin-independent receptor internalization. In our study, whether convallatoxin enhanced MOR endocytosis through a β-arrestin-2-dependent and AP2/clathrin-dependent mechanism, which warrants further investigation.

GPCR phosphorylation by GRKs^45–47^, protein kinases A and C (PKA and PKC)^48,49^, and c-Src^50^ contributes to receptor desensitization by preventing further coupling to G proteins. After phosphorylation, GPCRs may undergo internalization, dephosphorylation, resensitization and be recycled back to the plasma membrane. Morphine’s poor ability to induce MOR endocytosis represents that phosphorylated and desensitized receptors accumulate at the cell surface and cannot undergo the resensitization process described above^24^, which may explain why morphine-induced MOR phosphorylation is higher than full MOR agonist DAMGO-induced phosphorylation^51^. Our data show that morphine-induced MOR phosphorylation at serine 375 was attenuated by convallatoxin (Fig. 2C,E). We propose that convallatoxin may regulate MOR phosphorylation by promoting MOR endocytosis, thereby enabling increases in receptor dephosphorylation in the cytoplasm, which needs further verification.

We investigated the effects of convallatoxin on acute and chronic morphine in animal models of acute thermal and chronic pain. Convallatoxin significantly prevented the development of chronic morphine-induced tolerance and subsequent morphine withdrawal. Besides, acute convallatoxin administration reversed both morphine tolerance and withdrawal in morphine-tolerant mice (Fig. 4). In chronic pain, morphine partially relieved the mechanical allodynia (Fig. 6A). These results agree with previous studies showing that MOR agonists are highly effective in acute pain, but less so in chronic pain^52–54^. As compared to morphine, the combination of morphine and convallatoxin potently inhibited CFA-induced mechanical allodynia, which introduces the possibility that morphine’s poor ability to induce MOR endocytosis underlies its lack of efficacy in treating chronic pain. It is worth noting that convallatoxin only slightly reduced the potency, but not the magnitude, of morphine-induced antinociception and itself exerted no analgesia, and effects of convallatoxin were attenuated in the spinal AP2 or clathrin knockdown mice (Figs 5 and 6). Interestingly, the other two cardiac glycosides ouabain and digitoxin have been reported to regulate Na^+^/K^+^-ATPase activity, produce analgesia or antagonize morphine analgesia^55–57^. In contrast, rostafuroxin unable to inhibit Na^+^/K^+^-ATPase still enhanced morphine-induced MOR endocytosis. We thus suggest that other than Na^+^/K^+^-ATPase inhibition, convallatoxin potentially have a structure-activity relationship (SAR) related to ligand-induced MOR endocytosis which correlates with MOR-mediated analgesia. Therefore, it would be beneficial to modify the chemical structure of convallatoxin to increase ligand-induced MOR analgesia by enhancing ligand-induced MOR endocytosis and diminish cardiovascular side effects by reducing Na^+^/K^+^-ATPase inhibition.

## Conclusions

In conclusion, our findings demonstrate that convallatoxin as a novel enhancer of opioid-induced MOR endocytosis, which enhances opioid antinociception and decrease morphine tolerance and withdrawal. This provides new insights into clinical opioid-managed pain. The newly discovered regulatory mechanism of ligand-induced MOR endocytosis opens a new avenue to study MOR or other GPCR trafficking.

## Supplementary information

Supplementary information
