# Supplementary material for: Convallatoxin enhance the ligand-induced mu-opioid receptor endocytosis and attenuate morphine antinociceptive tolerance in mice
Source: Sci Rep. 2019 Feb 20;9:2405. doi: 10.1038/s41598-019-39555-x (PMC6382827; doi:10.1038/s41598-019-39555-x)

**Convallatoxin enhance the ligand-induced mu-opioid receptor endocytosis and attenuate morphine antinociceptive tolerance in mice**

Po-Kuan Chao^1^, Hsiao-Fu Chang^1^, Li-Chin Ou^1^, Jian-Ying Chuang^2^, Pin-Tse Lee^3^, Wan-Ting Chang^1^, Shu-Chun Chen^1^, Shau-Hua Ueng^1^, John Tsu-An Hsu^1^, Pao-Luh Tao^4^, Ping-Yee Law^5^, Horace H. Loh^5^, and Shiu-Hwa Yeh^1,2^ *

^1^ Institute of Biotechnology and Pharmaceutical Research, National Health Research Institutes, Zhunan, 35053, Taiwan

^2^ The PhD Program for Neural Regenerative Medicine, Taipei Medical University, Taipei, 110, Taiwan

^3^ Cellular Pathobiology Section, Intramural Research Program, National Institute on Drug Abuse, NIH/DHHS, Baltimore, MD 21224, USA

^4^ Center for Neuropsychiatric Research, National Heath Research Institutes, Zhunan, 35053, Taiwan

^5^ Department of Pharmacology, Medical School University of Minnesota, Minneapolis, MN 55455-0217, USA

*Correspondence to be addressed to:

Shiu-Hwa Yeh

Tel: (886)-37-246166 ext. 35759

Fax: (886)-37-586456

Email: [bau9763@nhri.org.tw](mailto:bau9763@bp.nhri.org.tw)

Present Address: Shiu-Hwa Yeh, Institute of Biotechnology and Pharmaceutical Research, National Health Research Institutes, Zhunan, 35053, Taiwan

**Supplemental Information**

**Supplementary Figures**

**
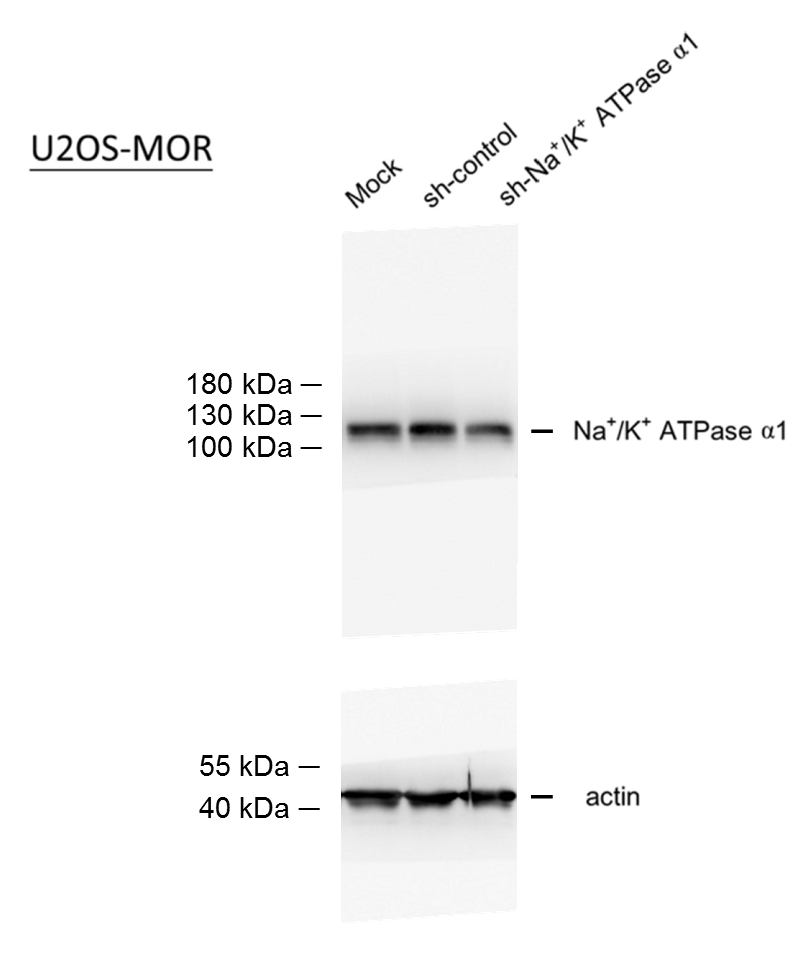
**

**Supplementary Figure 1.**

**Silencing of Na^+^/K^+^-ATPase α1 subunit in U2OS-MOR cells.** U2OS-MOR cells were transiently transfected with vehicle, sh-control or sh-Na^+^/K^+^-ATPase α1 to silence expression. The expression ofthe Na^+^/K^+^-ATPase α1 subunit in each treatment was determined using immunoblotting.


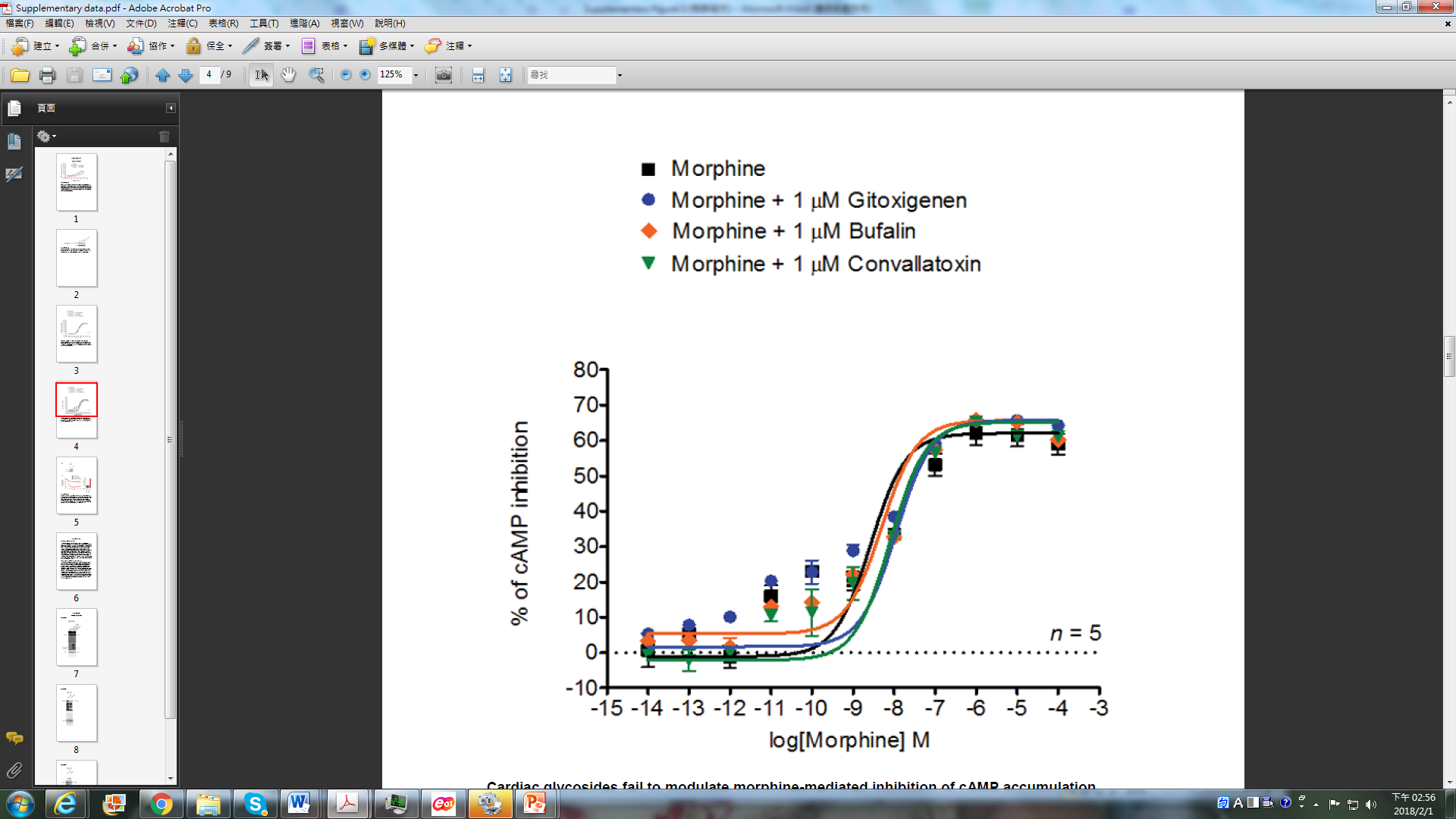


**Supplementary Figure 2.**

**Convallatoxin fail to modulate morphine-mediated inhibition of cAMP accumulation.** G protein coupling was measured by the inhibition of cyclic adenosine monophosphate (cAMP) accumulation in human embryonic kidney 293 cells expressing human mu-opioid receptor. Cells are treated with various concentrations of morphine in the absence or presence of gitoxigenen, bufalin, and convallatoxin. The values indicate the mean ± s.e.m.

**Supplementary Data**

**(full images of all gels or blots)**

**Figure 2C blots.**

**
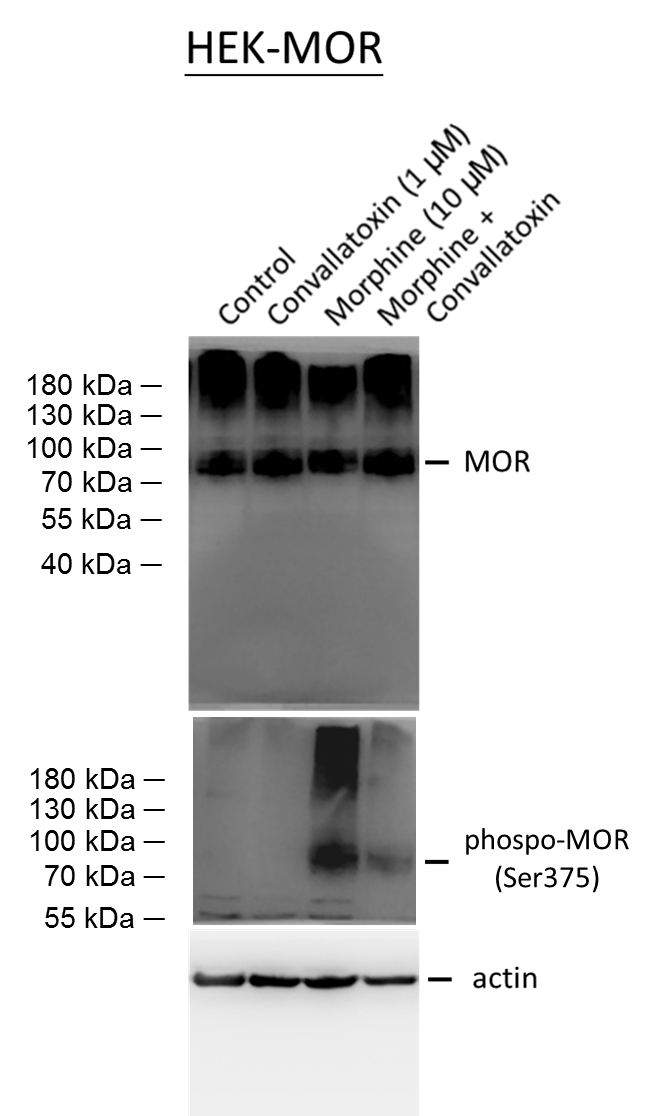
**

**Figure 3F blots.**


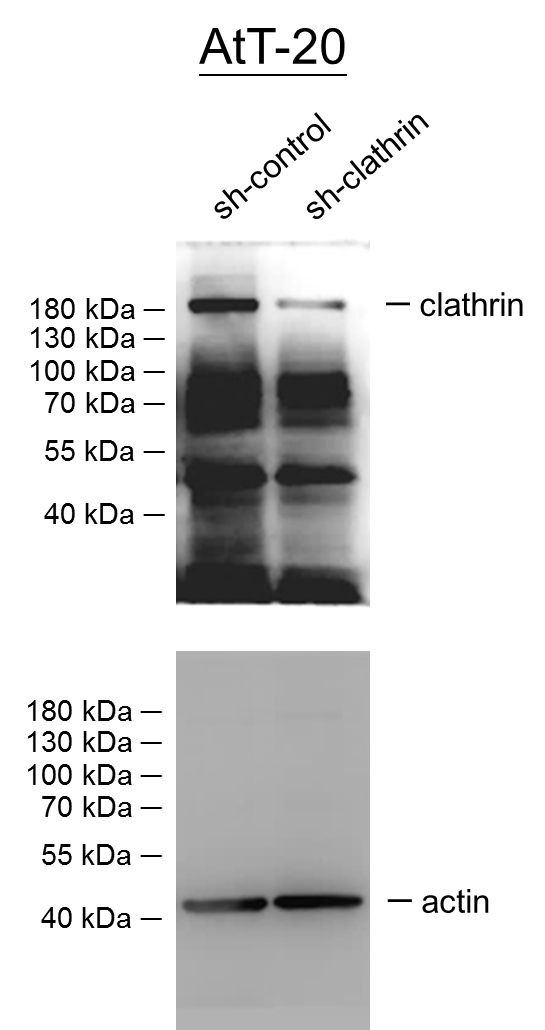


**Figure 3H blots.**


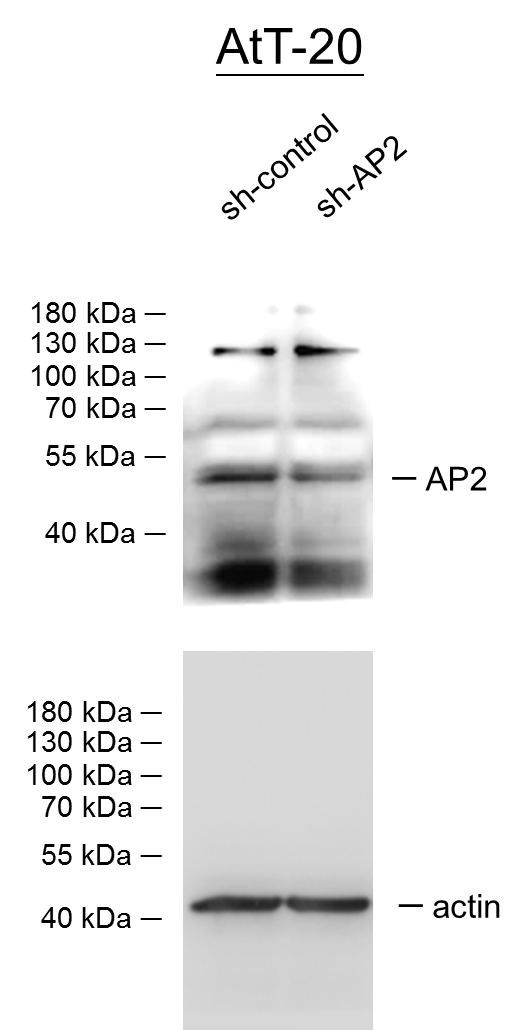

Supplement: Supplementary file 1 — Supplementary information [file 41598_2019_39555_MOESM1_ESM.docx]
